# Living Situation and Healthcare Utilization in Older Adults with Self-Reported Diabetes

**DOI:** 10.1093/geroni/igaf122.2461

**Published:** 2025-12-31

**Authors:** Amy Van Dusen, Rachel Bishop, Zane Deliu, Megan O’Mara, Carla Obeid, Asef Raiyan Hoque, Samantha Hahn

**Affiliations:** Central Michigan University College of Medicine, Byron Center, Michigan, United States; Central Michigan University, Mount Pleasant, Michigan, United States; Central Michigan University, Mount Pleasant, Michigan, United States; Central Michigan University, Mount Pleasant, Michigan, United States; Central Michigan University, Mount Pleasant, Michigan, United States; Central Michigan University, Mount Pleasant, Michigan, United States; Central Michigan University College of Medicine, Mount Pleasant, Michigan, United States

## Abstract

Diabetes is among the leading causes of morbidity and mortality in the United States, particularly in older adults. Prior research has demonstrated that social isolation is associated with worse diabetes outcomes, but the specific relationship between distinct living situations and healthcare utilization among older diabetic adults remains underexamined. This study aims to determine whether living situations, including living alone, living with a partner, or living with other adult(s), are associated with varying rates of hospitalizations, emergency department visits, and urgent care visits among older adults with diabetes. Data from the 2021 and 2022 National Health Interview Survey (NHIS) was analyzed via descriptive statistics, logistic regression, and zero-inflated negative binomial regression to investigate the relationship between the living situation and health outcomes as measured by overnight hospitalizations, number of urgent care visits, and emergency department visits of adults with diabetes over the age of 65. After adjusting for age, sex, race and ethnicity, education level, and comorbidities, participants living with a partner had significantly higher odds of visiting the emergency room when compared to individuals living alone. No significant differences were observed in urgent care visit frequency or hospitalization rates across living situations. This contrasts with the expectation that social isolation may drive increased healthcare utilization, suggesting that partners may encourage care-seeking behavior or results could reflect underlying differences in health status and access to outpatient care. Understanding factors contributing to increased ED visits is crucial for developing preventative measures to reduce the disease burden and financial burden associated with diabetes.

